# Antioxidant but not ergogenic or altitude-protective: a meta-analysis of Rhodiola species in healthy adults

**DOI:** 10.3389/fphys.2026.1908897

**Published:** 2026-10-07

**Authors:** Jie Mou, Jingxian Yin, Lingting Fan, Wen Zhang, Junxian He, Hongyu Sun

**Affiliations:** 1 College of Medicine, Southwest Jiaotong University, Chengdu, China; 2 Center for Animal Experimentation, Clinical Biobank, and Bioinformatics, The General Hospital of Western Theater Command, Chengdu, China

**Keywords:** antioxidant capacity, meta-analysis, plateau acclimatization/deacclimatization, Rhodiola rosea, Rhodiola species

## Abstract

**Background:**

Plants of the genus *Rhodiola* (including *R. rosea* and *R. crenulata*) is purported to enhance antioxidant capacity and altitude adaptation, yet clinical evidence remains conflicting. This meta-analysis evaluates its effects on systemic antioxidant markers and altitude adaptation outcomes in healthy adults.

**Methods:**

Nine Randomized Controlled Trials (RCTs, n=424) were identified from PubMed, Cochrane, Medline, and China National Knowledge Infrastructure (CNKI). Primary outcomes included maximal oxygen uptake (VO_2_max), total antioxidant capacity (TAC), thiobarbituric acid reactive substances (TBARS), malondialdehyde (MDA), and acute mountain sickness (AMS) incidence. Data were pooled using RevMan 5.4 with random/fixed-effects models.

**Results:**

R. rosea significantly increased TAC (P=0.02) and showed a trend toward reducing TBARS levels (P=0.06), indicating enhanced systemic antioxidant capacity. However, it showed no significant effect on VO_2_max (95% CI: -0.91 to 2.99; P=0.30) or MDA levels (P=0.07). Crucially, R. rosea did not prevent AMS (OR=1.02, 95% CI: 0.69–1.52) but mitigated some symptoms of high-altitude deacclimatization (P<0.05).

**Conclusion:**

Extracts of Rhodiola species significantly improves systemic antioxidant status and may alleviate high-altitude de-adaptation symptoms. However, current evidence does not support its efficacy for improving VO_2_max, reducing MDA levels, or preventing AMS. These uncertainties likely stem from heterogeneous study designs, sample sizes, variable dosing, and inconsistent intervention durations.

**Systematic review registration:**

https://www.crd.york.ac.uk/PROSPERO/view/CRD420251004228, identifier CRD420251004228.

## 1 Introduction

Rhodiola rosea (R. rosea), also known as roseroot, is a perennial plant that grows in high-altitude mountainous regions of Asia, Europe, and North America (Pu et al., 2020). Rhodiola rosea contains a variety of active components, including phenylpropanoid compounds (such as rosavin, rosin, and rosarin), phenolic compounds (such as salidroside and p-tyrosol), flavonoids, and organic acids (such as gallic acid), which endow Rhodiola rosea with a range of biological activities (Panossian et al., 2010). It is noteworthy that Rhodiola crenulata (R. crenulata), another species within the same genus, shares similar traditional uses but differs from R. rosea in chemical composition—particularly in the relative abundance of salidroside and rosavin—and may therefore exhibit distinct pharmacological profiles. Notably, polysaccharides also constitute an important class of bioactive constituents in R. rosea, yet their contribution to antioxidant activity has received limited attention. In medicinal and edible fungi such as Poria cocos and Ganoderma lucidum, polysaccharides have been systematically shown to exert immunomodulatory, anti-inflammatory, and antioxidative stress effects through multi-targeted pathways (Xie et al., 2025). By extension, the polysaccharide fraction of R. rosea may similarly participate in its overall antioxidant defense, complementing the actions of phenolic compounds. This multi-component antioxidant profile is not unique to R. rosea. Similar synergistic mechanisms have been described in other medicinal and edible plants; for instance, the systematic review by Wu et al. demonstrated that Phyllanthus emblica Linn. exerts protective effects against oxidative stress-induced aging through the coordinated actions of polyphenols and polysaccharides, which scavenge free radicals, modulate redox-sensitive signaling pathways, and reinforce endogenous antioxidant defenses (Wu et al., 2025). By analogy, the diverse phenolic and polyphenolic constituents of R. rosea may confer antioxidant benefits through comparable, multi-targeted pathways.

The antioxidant effects of R. rosea are mainly attributed to its abundant phenolic compounds and other antioxidant substances (Tinsley et al., 2024). Studies have shown that extracts of R. rosea exhibit antioxidant potential against human red blood cells *in vitro*, preventing oxidative damage caused by hypochlorous acid (Chen et al., 2022; Battistelli et al., 2005; De Sanctis et al., 2004). Additionally, high doses of R. rosea extracts may alter the shape of red blood cells without oxidative stress (Qian et al., 2011). Some experimental studies also suggest that R. rosea may be beneficial for athletes, as it may improve work capacity and reduce changes in inflammatory parameters (such as C-reactive protein) and skeletal muscle damage markers (such as creatine kinase) caused by intense exercise (Ivanova Stojcheva and Quintela, 2022; Lu et al., 2022; Skarpanska-Stejnbron et al., 2009).

The antioxidant properties of R. rosea may help alleviate oxidative stress caused by stressors such as exercise or high-altitude activities, which is particularly important after intense exercise and high-altitude activities, as these situations generate more reactive oxygen species (ROS) and deplete antioxidants (Chen et al., 2014; Lo et al., 2018; Sanz-Barrio et al., 2023; Todorova et al., 2021). Consistent with the broader paradigm observed in P. emblica and related species, wherein combined polyphenolic and polysaccharide fractions produce superior antioxidant outcomes than isolated compounds alone, the multi-component nature of R. rosea suggests that its stress-protective effects likely arise from integrated rather than single-molecule actions. This view is further supported by evidence from fungal polysaccharide research, where the biological activity of Poria cocos and Ganoderma polysaccharides is closely linked to their structural diversity and monosaccharide composition, underscoring that the antioxidant potential of complex natural products depends on the concerted contribution of multiple chemical classes rather than any single constituent (Xie et al., 2025). Rhodiola rosea may function by stimulating metabolism, promoting the utilization of fatty acids, improving the body’s resistance to physical fatigue, and providing cardiac protection (Hou et al., 2024; Iannuzzo et al., 2024; Yang et al., 2024). Although the antioxidant and adaptogenic effects of Rhodiola rosea have been confirmed in some animal models, evidence of these effects in humans remains limited and inconsistent.

Additionally, as more people engage in activities at high altitudes, to adapt to high-altitude environments or to cope with various clinical symptoms associated with ascending to high altitudes (Gatterer et al., 2024), individuals often opt for Rhodiola rosea preparations (Chen et al., 2014). However, there is still a lack of evidence-based research to confirm their efficacy and safety. Therefore, in this article, we conduct a meta-analysis of RCT studies focusing on the antioxidant capabilities of R. rosea.

## 2 Methods

### 2.1 Protocol registration

We conducted this review and reported the results following the guidelines recommended by the Cochrane Handbook for Systematic Reviews of Interventions and the Preferred Reporting Items for Systematic Reviews and Meta-analyses (PRISMA) statement (Page et al., 2021). This protocol has already been registered on Prospero (CRD420251004228).

### 2.2 Eligibility criteria

The inclusion criteria were as follows: (1) RCTs evaluating the effects of Rhodiola rosea extract on antioxidation and high-altitude adaptation were included, without restrictions on language or publication status. (2) Participants: Subjects were healthy adults, or individuals who were about to ascend from low-altitude areas to high altitudes, who had not taken any antioxidative preparations or any preventive medications or herbs within one month prior to the study, and had no history of high-altitude residence or high-altitude acclimatization training experience. There were no restrictions on the age, gender, or ethnicity of the subjects. (3) Intervention: The intervention for the treatment group was oral administration of Rhodiola rosea preparations, including both single-component and compound formulations. The control group received a placebo. (4) Outcomes: This review included studies that reported predefined outcome measures.

The exclusion criteria were as follows: (1) duplicate articles; (2) full texts of publications that cannot be obtained, or studies that do not report basic information about subjects or intervention measures; (3) articles that do not report complete outcomes; (4) studies involving non-healthy adults.

### 2.3 Search strategy

We searched PubMed, the Cochrane Library, Medline and CNKI up to March 29, 2025. The search terms/keywords used were (“Adaptation, Biological”[Mesh] OR “Oxygen”[Mesh] OR “Reactive Oxygen Species”[Mesh] OR “Biological Oxygen Demand Analysis”[Mesh] OR “Singlet Oxygen”[Mesh] OR “Oxygen Consumption”[Mesh] OR “Oxygen Saturation”[Mesh] OR “Altitude Sickness”[Mesh] OR “Hypoxia”[Mesh] OR “Hypoxia-Ischemia, Brain”[Mesh] OR “Cell Hypoxia”[Mesh] OR “Hypoxia, Brain”[Mesh]) AND (“Rhodiola”[Mesh]) AND “RCT” [Mesh], with adjustments made for the use of different databases.

### 2.4 Study selection and data extraction

Two authors independently conducted literature searches and screenings based on the search strategy and eligibility criteria. After the eligible studies were ultimately included, the authors continued to independently extract data using a pre-designed form, which included citation, sample size, participants, interventions, outcome measures, results, and information related to the study design.

### 2.5 Risk of bias assessment

Two authors independently assessed the risk of bias in individual RCTs using the Cochrane Risk of Bias Tool, which includes the following domains: (1) the randomization process; (2) allocation concealment; (3) blinding of participants and personnel; (4) whether predefined outcomes were fully reported; (5) blinding of outcome assessment; (6) incomplete outcome data; (7) other biases. Each domain was judged as having a low risk of bias, some concerns, or a high risk of bias. Finally, an overall assessment of the risk of bias for each study was made based on the results of the above five domains. Two authors (MJ, FLT) independently assessed the risk of bias and cross-checked their findings, with any discrepancies resolved by consensus or negotiation with other author.

### 2.6 Statistical analysis

The meta-analysis was conducted using RevMan 5.4 software (Nordic Cochrane Centre, Cochrane Collaboration, Copenhagen, Denmark). Continuous data were expressed as the mean difference with standard deviation for effect size. Meta-analysis was performed when at least two RCTs in the treatment and control groups used the same intervention and reported the same outcomes. The analysis model was determined based on the results of the heterogeneity test. When the heterogeneity test indicated I^2^ > 50%, a random-effects model was used; otherwise, a fixed-effects model was applied (Higgins et al., 2003). Subgroup analyses of the primary outcomes were conducted based on the following factors: (1) the intervention treatment preparation being Rhodiola rosea monotherapy or compound formulation; (2) the dosage of the intervention treatment; (3) the duration of the intervention treatment; (4) regular exercisers versus non-exercisers among healthy subjects; (5) the year of publication. The quality of evidence for the primary outcomes was assessed using the Grading of Recommendations Assessment, Development and Evaluation (GRADE) criteria. When at least ten RCTs were combined in a meta-analysis, funnel plot analysis was performed to detect publication bias.

## 3 Results

### 3.1 Search outcomes

Initially, a total of 124 articles were identified from four databases. After excluding 105 duplicate publications, 7 articles were further excluded after reviewing the titles and abstracts. We then downloaded 12 articles from the databases for full-text screening. Based on the eligibility criteria, 3 articles were excluded. Ultimately, 9 RCTs were included. The PRISMA flowchart for the literature selection process is shown in **Figure 1**.

**Figure 1:**
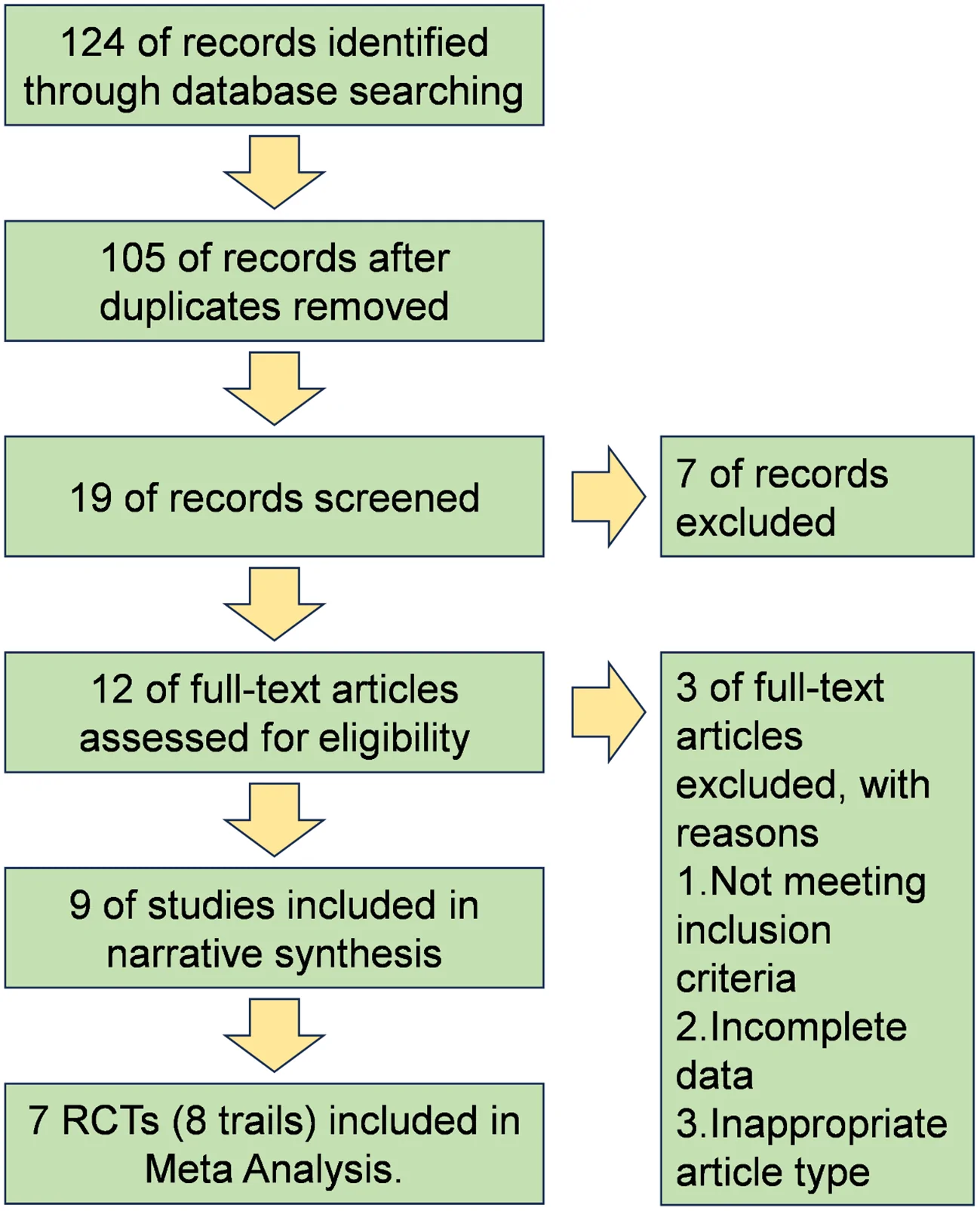
PRISMA flow diagram illustrating the literature search and screening process of this systematic review and meta-analysis.

### 3.2 Characteristics of included studies

The nine articles included, with eight in English and one in Chinese. The total sample size was 424 participants. All subjects were healthy adults who had not exercised or taken any antioxidative preparations or traditional Chinese medicine formulations prior to the study, nor had they lived at high altitudes. The occupations of the study subjects included college students, volunteers, soldiers, athletes, and the general population. Interventions in the studies included Rhodiola rosea monotherapy and combined formulations, with the control group receiving placebo treatment. The duration of treatment ranged from several hours to 8 weeks. In three RCTs, Rhodiola rosea preparations were taken continuously for 4 weeks at dosages of 170mg/day, 100mg/day, and 200mg/day, respectively; one study involved continuous intake for 1 week at a dosage of 1600mg/day and 800mg/day; one study for 2 weeks at a dosage of 200mg/day; one study for 7 weeks at a dosage of 1080mg/day; and one study for 8 weeks at a dosage of 1400mg/day (**Table 1**).

**Table 1:** Basic information about the study participants.

Research information	Sample size	Sex (% male)	Sample characteristics	Research area	Duration of intervention	Intervention formulation	Dose (mg/d)	Comparison
Yi-Hung Liao, 2019	14	57%	Healthy adults	China	8 weeks	Rhodiola Rosea/Cordyceps	1400	placebo-controlled study
Chung-Yu Chen, 2014	18	100%	Sportsmen	China	2 weeks	Rhodiola Rosea/Cordyceps	200	placebo-controlled study
Te-Fa Chiu, 2013	120	47%	Healthy adults	China	1 week	Rhodiola rosea	800	placebo-controlled study
Zi-fu Shi, 2011	130	100%	Healthy adults	China	15 days	Rhodiola rosea	2000	placebo-controlled study
A Parisi, 2010	14	100%	Sportsmen	Italian	4 weeks	Rhodiola rosea	170	void
ZHANG Zhang-jin 2009	67	100%	Sportsmen	China	7 weeks	Rhodiola Rosea/Ginkgo Biloba	1080	placebo-controlled study
Ana Skarpanska-Stejnborn, 2009	22	100%	Sportsmen	Polish	4 weeks	Rhodiola rosea	100	placebo-controlled study
Katrien De Bock, 2004	24	100%	Healthy adults	Belgian	4 weeks	Rhodiola rosea	200	placebo-controlled study
Stacie L Wing, 2003	15	40%	Healthy adults	America	1 week	Rhodiola rosea	1600	placebo-controlled study

### 3.3 Methodological quality assessment

**Figure 2** shows the overview of the risk of bias assessment for all included trials. Among the seven domains in the Cochrane Risk of Bias assessment tool, the risk of bias in the randomization process was considered low in seven studies that reported methods of random sequence generation, eight studies reported allocation concealment, and three studies reported blinding of participants and personnel as low risk; one additional study reported blinding of outcome assessment as low risk. Moreover, all studies showed low risk for selective reporting of outcome data; the rest were assessed as unclear. Based on the description in the methods section of the articles, there was no evidence of selective reporting of results. Therefore, the domain of reporting bias is considered at low risk.

**Figure 2:**
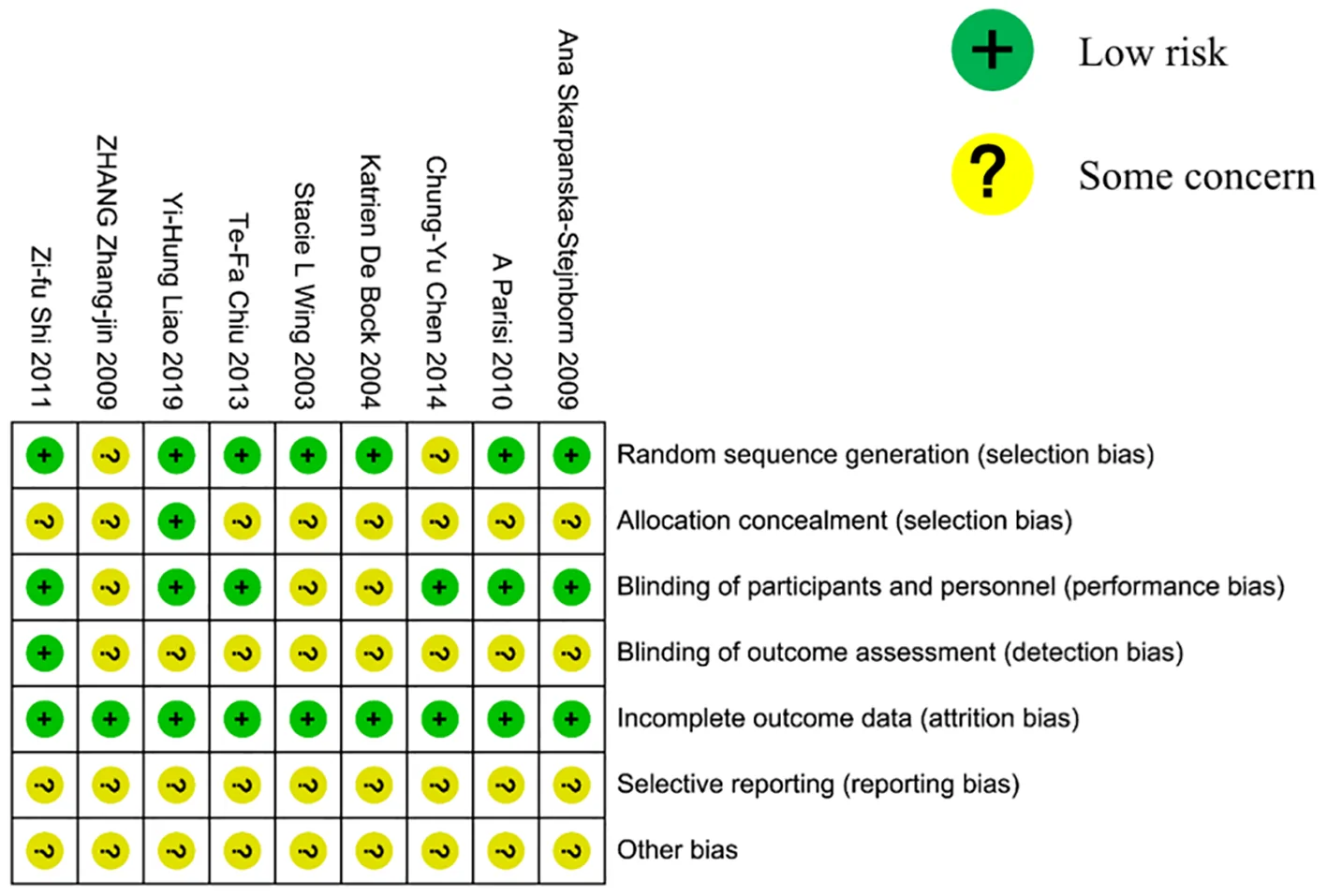
Cochrane risk of bias summary.

### 3.4 Outcome evaluation

#### 3.4.1 Maximum oxygen uptake (VO2 max)

The effect of long-term Rhodiola rosea supplementation on the maximum oxygen uptake of healthy adults compared to a placebo was assessed in six studies. The meta-analysis results indicated that after 2 to 8 weeks of Rhodiola rosea supplementation, the VO2 max of the experimental group was higher than that of the placebo group, with a 95% CI (Confidence Interval) ranging from -0.91 to 2.99, but the difference was not statistically significant (P=0.30) (**Figure 3**). It is worth noting that among these studies, five of them, with four experiments, showed that the Rhodiola rosea group significantly improved oxygen uptake compared to the placebo group (De Bock et al., 2004; Schwarz et al., 2024; Liao et al., 2019; Parisi et al., 2010; Chen et al., 2014; Zhang et al., 2009).

**Figure 3:**
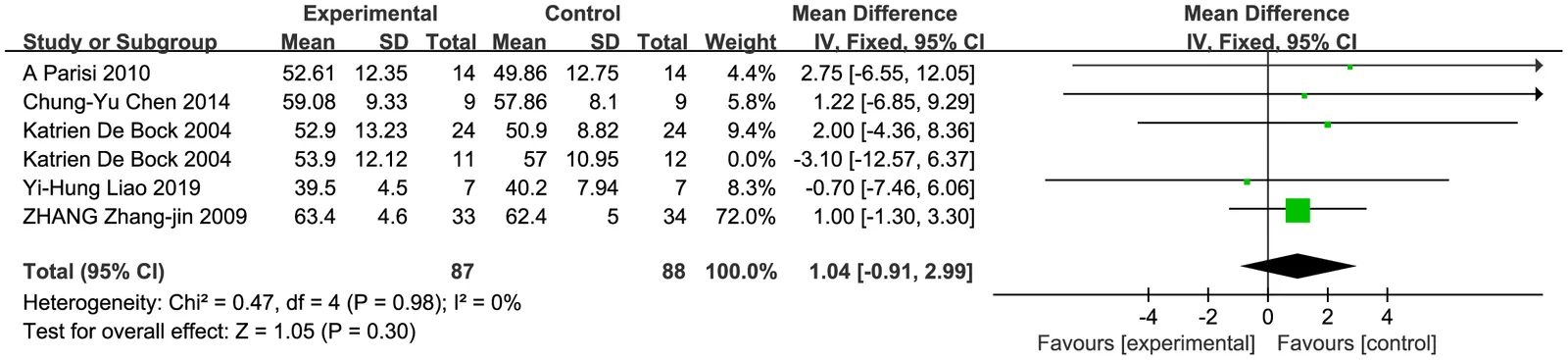
Effect of Rhodiola rosea on maximal oxygen uptake in healthy adults (Unit: mL/kg/min).

#### 3.4.2 Total antioxidant capacity

TAC is a measure of the combined capabilities of all antioxidant substances in a biological sample. In two studies, the effect of Rhodiola rosea on the antioxidant capacity of sedentary healthy adults and trained athletes compared to a placebo group was assessed. The meta-analysis results indicated that the TAC in the Rhodiola rosea group was significantly higher than that in the placebo group (P=0.02) (Liao et al., 2019; Skarpanska-Stejnbron et al., 2009) (**Figure 4**).

**Figure 4:**
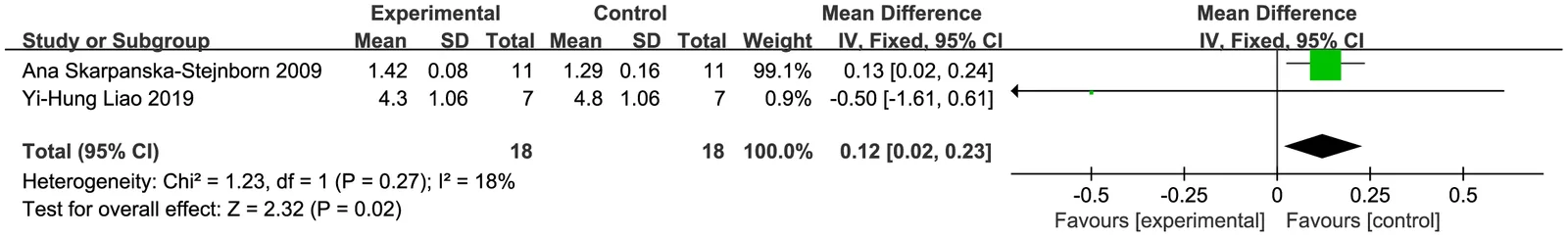
Effect of Rhodiola rosea on TAC in healthy adults (Unit: mmol TE/L).

#### 3.4.3 Thiobarbituric acid reactive substances

Studies have shown that when the human body is subjected to stress, such as exercise, emotional changes, or environmental alterations, it can lead to metabolic disorders, especially in lipid metabolism (Yaribeygi et al., 2017). When the body’s oxygen demand increases, lipids are more prone to oxidation, which can subsequently damage cells and organs. TBARS (Thiobarbituric Acid Reactive Substances) is a biochemical indicator used to assess the degree of lipid peroxidation and is primarily employed to detect the level of oxidative stress in biological samples. Two studies have indicated that Rhodiola rosea is beneficial in reducing the production of TBARS under stress conditions (p=0.06). A randomized controlled trial demonstrated that under stressful conditions, the level of TBARS decreased from 2.8 ± 0.73 to 2.3 ± 0.38 after treatment with salidroside (a compound found in Rhodiola rosea). These results suggest that salidroside is advantageous in improving oxidative stress under stressful conditions (**Figure 5**).

**Figure 5:**
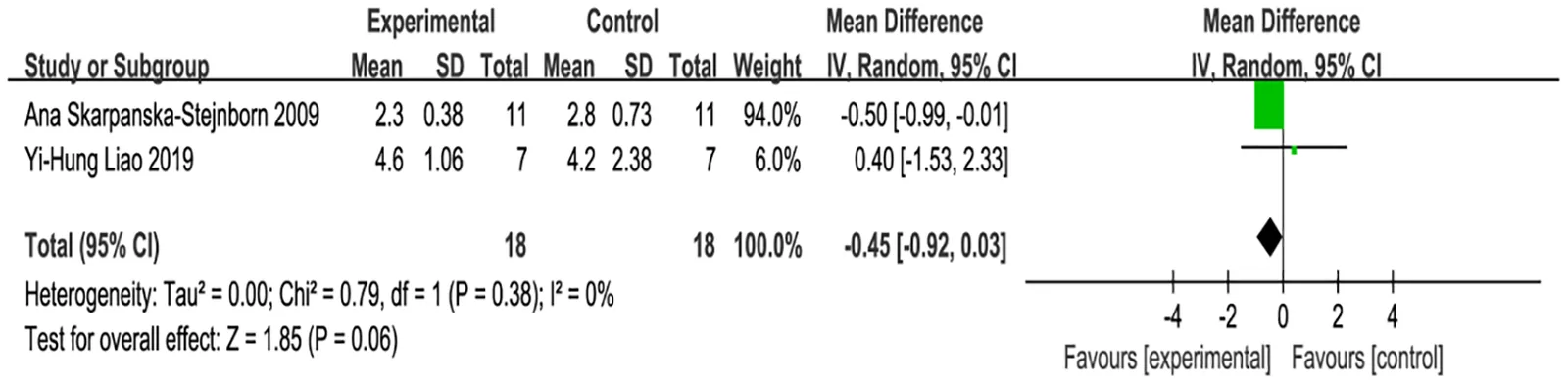
Effect of Rhodiola rosea on TBARS in healthy adults (Unit: nmol/L).

#### 3.4.4 Malondialdehyde

As a end product of lipid peroxidation, elevated levels of MDA are typically associated with increased oxidative stress. In two studies, the effect of Rhodiola rosea on the antioxidant capacity of healthy adults compared to a placebo was assessed. The meta-analysis results showed that after hypoxic stress or stress training, the MDA levels in the Rhodiola rosea group were comparable to those in the placebo group, with no statistically significant difference (P=0.07) (Parisi et al., 2010; Wing et al., 2003) (**Figure 6**).

**Figure 6:**
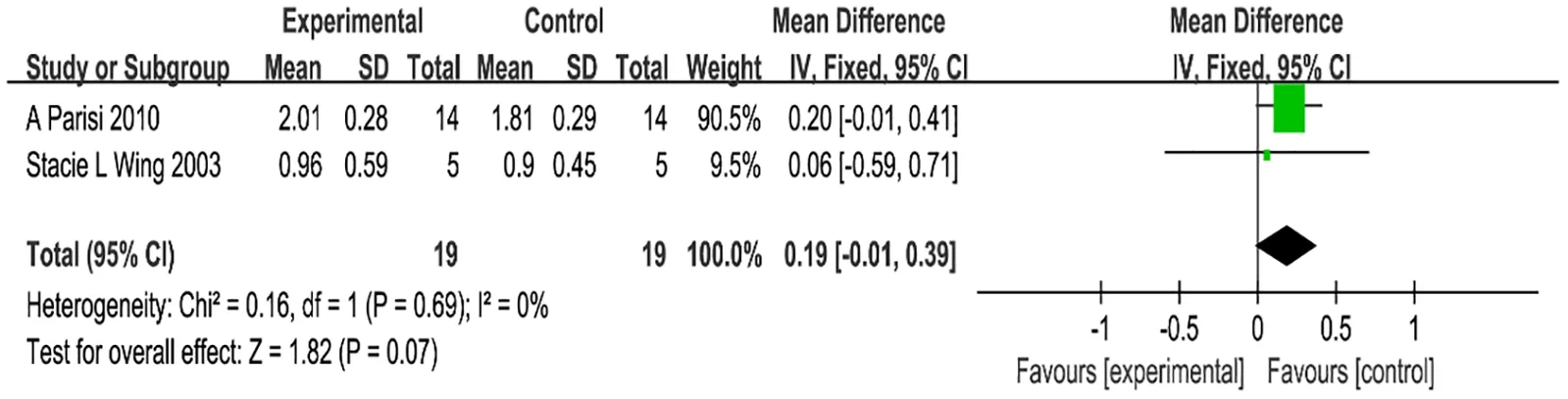
Effect of Rhodiola rosea on malondialdehyde in healthy adults (Unit: nmol/L).

#### 3.4.5 Subgroup analysis

In this study, as shown in **Figure 7**, we conducted an analysis of VO_2_max across various subgroups to assess the impact of different interventional measures on maximal oxygen uptake. The results of this meta-analysis indicate that the effects of the interventions across the different subgroups on VO_2_max are not significant. These findings suggest that, although Rhodiola rosea and its combined formulations have shown potential health benefits in some studies, their effect on enhancing maximal oxygen uptake remains unclear. Further research is needed to explore the potential impact of different interventional measures on VO_2_max.

**Figure 7:**
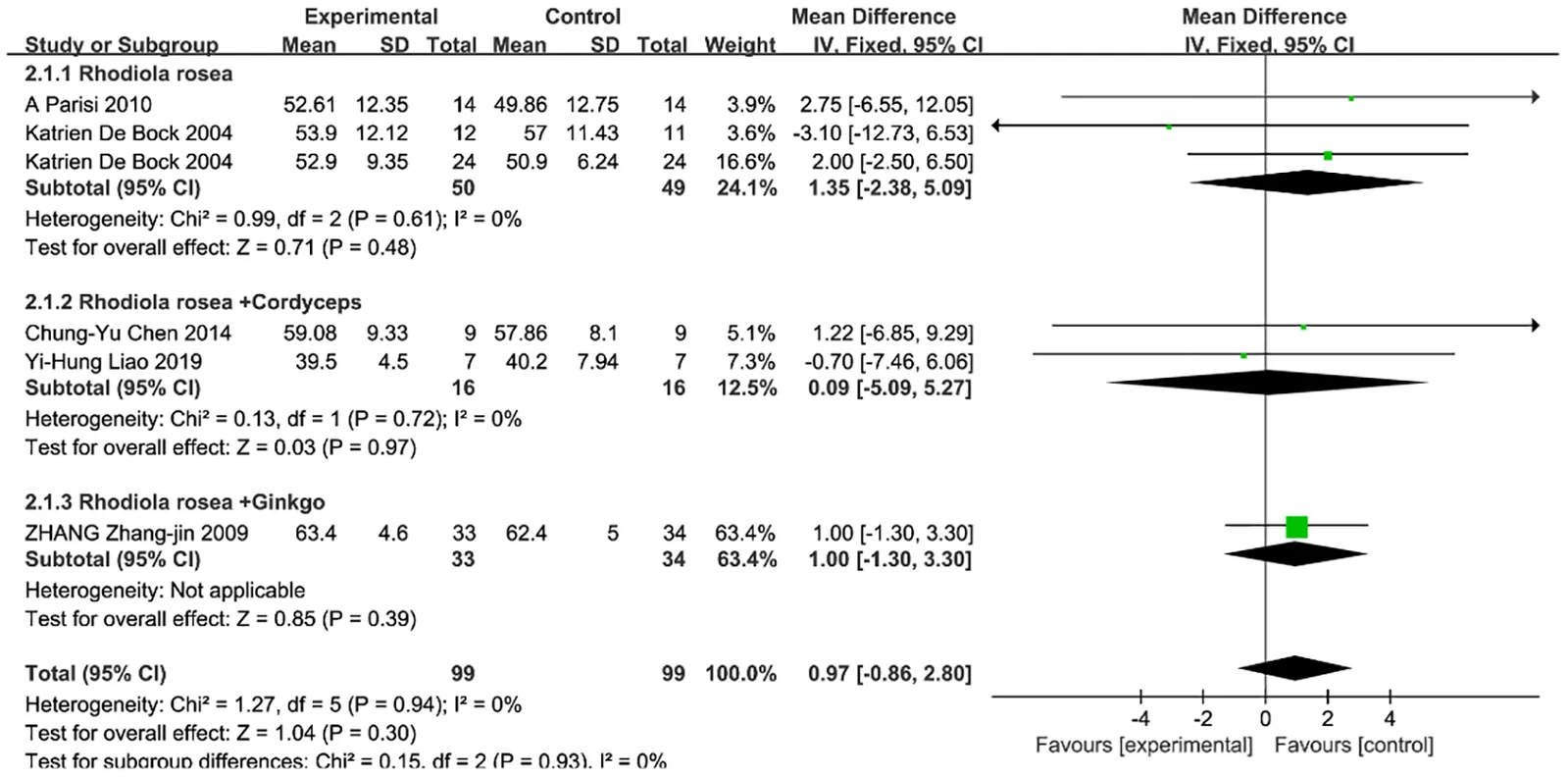
Maximum oxygen Uuptake about the subgroup (Unit: mL/kg/min).

### 3.5 The impact of Rhodiola rosea on plateau adaptation

#### 3.5.1 The efficacy of Rhodiola rosea in preventing acute mountain sickness

Rhodiola crenulata, commonly referred to as “Hong Jing Tian” in Chinese and distinct from R. rosea in its higher salidroside-to-rosavin ratio, has been extensively utilized in the Himalayan regions of Nepal and Tibet for the prevention of acute mountain sickness (AMS). However, its efficacy had not been scientifically validated prior to this study. A randomized, double-blind, placebo-controlled crossover trial was conducted, enrolling local Chinese adults aged 23 to 55 years, residing at altitudes of 250m or below, excluding individuals who did not meet the inclusion criteria (e.g., unwilling to accept the study protocol, with specific medical histories, etc.). Participants were randomly assigned to two treatment sequences, receiving either Rhodiola rosea extracts or placebo, at a dosage of 800 mg daily for 7 days, administered 2 days before mountain climbing, and crossed over to the alternative treatment after a 3-month washout period. Participants rapidly ascended from an altitude of 250 to 3421m, with trials conducted in December 2010 and April 2011.A total of 102 participants completed the trial, with no demographic differences between the rhodiola rosea group and the placebo group. There was no significant difference in the incidence of AMS between the R. crenulata extract group and the placebo group (both 60.8%; adjusted odds ratio OR=1.02, 95% confidence interval CI=0.69-1.52). The Rhodiola crenulata extract group and placebo group had AMS incidences of 35.3% and 29.4%, respectively (OR=1.42, 95% CI=0.90-2.25). The results of this study indicate that Rhodiola crenulata extract is no more effective than placebo in preventing acute mountain sickness and should not be recommended as a preventive measure for AMS. These findings are consistent with the results of our aforementioned study on the antioxidant capacity of Rhodiola species in healthy adults (Chiu et al., 2013).

#### 3.5.2 The impact of Rhodiola rosea on high-altitude de-adaptation syndrome

With the increased frequency of military operations at high altitudes, there has been a significant rise in the number of individuals experiencing high-altitude de-adaptation symptoms upon return to lower altitudes from high-altitude missions, severely affecting the physical and mental health of military personnel and the maintenance of troop combat effectiveness [18]. A study was conducted to explore the interventional effects of a traditional Chinese medicine compound formula on high-altitude de-adaptation syndrome, providing a scientific basis for the treatment of high-altitude de-adaptation syndrome in individuals returning from high-altitude areas to lower altitudes. The study selected military personnel who experienced high-altitude de-adaptation symptoms after returning to lower altitudes following high-altitude earthquake relief operations from April 14 to August 7, 2010, as subjects for observation. A randomized, single-blind, placebo-controlled trial design was employed, randomly assigning 534 military personnel into three groups: the Rhodiola capsule group, the ginsengma pollen capsule group, the San Kang capsule group, and the placebo group, each receiving corresponding medication for 15 days. Self-assessment questionnaires for high-altitude de-adaptation symptoms were used to evaluate the treatment efficacy based on symptom improvement rates and inter-group differences in symptom improvement. Ultimately, 543 military personnel were included in the statistical analysis, with no significant differences among all groups in terms of age, health status, labor intensity, training duration, and high-altitude duty time. All three medications showed improvement effects on high-altitude de-adaptation symptoms, with significant symptom reduction compared to the placebo group (P < 0.05). Among them, compared to the placebo, the Rhodiola capsule primarily alleviated symptoms such as fatigue, somnolence, chest tightness, palpitations, dizziness, lack of concentration, and memory decline (Shi et al., 2011) (**Figure 8**). These findings are contradictory to our current observations and require further validation through experiments with larger sample sizes.

**Figure 8:**
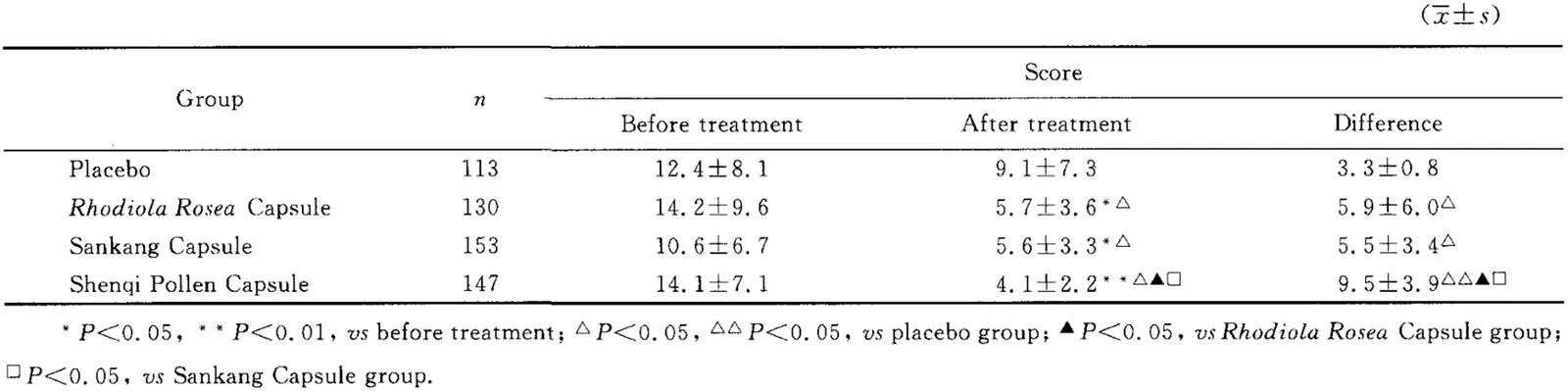
Scores of high-altitude de-adaptation syndrome before and after treatment among various military groups upon return to lower altitudes.

## 4 Discussion

### 4.1 Main findings

This study encompasses nine randomized controlled trials with a total sample size of 424 participants. Overall, the study demonstrates that, under stress conditions, Rhodiola rosea or its extracts significantly improve oxidative stress indicators compared with placebo. Additionally, it may enhance the body’s maximum oxygen uptake to combat stress-induced damage, although further experimental validation is required. This suggests that the stress-mitigating effects of Rhodiola rosea may be attributed to its potent antioxidant properties. To further elucidate the oxygen-enhancing capacity of Rhodiola rosea, we conducted subgroup analyses of the maximum oxygen uptake in healthy adults based on intervention formula, dosage, participants, and publication year. The results showed no statistically significant differences between the Rhodiola rosea group and the placebo group (P>0.05). We have indicated that combinations of Rhodiola rosea with Cordyceps sinensis/Ginkgo biloba, higher dosages (1000–1600 mg/day) of Rhodiola rosea supplements, longer intervention periods, and adult athletes/regular exercisers are conducive to maximum oxygen uptake. Nevertheless, given the small sample sizes of the studies, further validation with larger samples is warranted.

Additionally, Rhodiola species, particularly R. crenulata as a natural Tibetan medicine, play a significant role in high-altitude diseases. In terms of acute mountain sickness (AMS), studies have shown that Rhodiola extracts—primarily from R. crenulata—are no more effective than placebo in preventing AMS which is consistent with the current acute mountain guidelines (Luks et al., 2024). High-quality research is needed for further validation. Another randomized, single-blind, placebo-controlled trial indicated that Rhodiola rosea helps to alleviate symptoms of high-altitude maladaptation, showing significant efficacy in relieving fatigue, somnolence, chest tightness, palpitations, dizziness, lack of concentration, and memory impairment. However, the research findings are limited. This differential efficacy may be partially attributed to the metabolic-regulatory properties of Rhodiola constituents. A recent review identified flavonoids, triterpenes, alkaloids, and polysaccharides as major active substances in food and medicinal homological resources that regulate lipid metabolism primarily through AMPK and PPARγ pathways (Hao et al., 2024); the flavonoids, phenylpropanoids, and polysaccharides abundant in R. rosea share mechanistic similarities with these constituents. Such metabolic modulation may be more relevant to the restoration of homeostasis during high-altitude de-adaptation than to the acute hypoxic vasoconstriction underlying AMS, thereby explaining the observed discrepancy in clinical outcomes.

Among the nine articles included in this study, all employed single-blind or double-blind placebo-controlled designs. Thus, the overall methodological quality was fair, but with certain risks of bias. For studies on Rhodiola species’ promotion of oxygen uptake and high-altitude adaptation, due to a relatively large number of studies and sample sizes, the results among studies were consistent, therefore, GRADE evidence was rated as high-level evidence, indicating good reliability and stability of the results. For studies on the impact of Rhodiola species on oxidative stress indicators, due to the assessment using smaller sample sizes, the results were influenced by various biases, and further evaluation with high-quality studies is needed.

### 4.2 Strengths and limitations of this study

Systematic evaluations of Rhodiola species’ impact on healthy adults’ oxygen uptake capacity, antioxidant indicators, and high-altitude adaptation are scarce, with existing research focusing mainly on single studies. Building on previous literature, we conducted a comprehensive search of all RCTs comparing Rhodiola crenulata with placebo, merging RCTs with identical treatments, control interventions, and outcome indicators into meta-analyses, and performing other descriptive analyses. Thus, this review introduces the overall evidence of Rhodiola crenulata in terms of oxygen uptake capacity, antioxidant capacity, prevention of acute mountain sickness, and treatment of high-altitude de-adaptation. Moreover, we conducted subgroup analyses of participants’ oxygen uptake capacity based on intervention formulation, dosage, intervention duration, subjects, and publication year to identify specific populations that may benefit from Rhodiola crenulata’s oxygen-enhancing capacity.

This study has certain limitations; participants were primarily young males, thus the generalizability of the results to other age groups and female populations may be limited. Additionally, the source and dosage forms of Rhodiola rosea extracts varied across studies, and this heterogeneity in intervention measures may lead to differences in study outcomes. Furthermore, the methods for assessing maximum oxygen uptake differed, potentially causing heterogeneity in meta-analyses. However, to minimize potential impacts on the results, we included only values measured immediately after exercise.

### 4.3 Impact on clinical practice and research

In clinical practice, the findings suggest that Rhodiola rosea may serve as an adjunct antioxidant supplement for healthy adults exposed to oxidative stressors—specifically, individuals undergoing intense physical training, rapid altitude ascent, or high-stress occupational demands—at dosages of 200–600 mg/day of standardized extract (containing ≥3% rosavins and ≥1% salidroside) over intervention periods of at least 4 weeks (Liao et al., 2019; Skarpanska-Stejnbron et al., 2009). However, given the absence of significant effects on MDA and VO_2_max, it should not be relied upon as a standalone ergogenic aid or acute hypoxic protectant. Caution is warranted in extrapolating these findings to non-exercising populations, females, or individuals over 50 years of age, as these demographics were underrepresented in the included trials. Moreover, the research on Rhodiola rosea’s improvement of high-altitude de-adaptation symptoms lacks more robust clinical evidence, and more high-quality studies are recommended for confirmation. Notably, subgroup analyses indicated that a combination with Cordycepsis sinensis/Ginkgo biloba leaves, longer intervention periods, higher doses, and regular exercise may contribute to enhancing the body’s oxygen uptake capacity.

Future studies should prioritize the following specific directions: (1) Standardizing botanical material by specifying the *Rhodiola* species (*R. rosea* versus *R. crenulata*), extraction method, and phytochemical standardization (rosavin and salidroside content); (2) Defining dosage ranges through dose–response trials, with particular attention to thresholds above 1000 mg/day where ergogenic effects may emerge; (3) Expanding participant diversity by including female subjects, middle-aged and elderly adults (≥50 years), and sedentary versus trained cohorts to clarify population-specific efficacy; (4) Extending intervention duration to ≥8 weeks to assess whether prolonged supplementation can translate improved TAC into measurable reductions in MDA or enhanced VO_2_max; and (5) Incorporating mechanistic biomarkers such as lipid profiles, glucose homeostasis indicators, and adipokine levels alongside traditional antioxidant endpoints to clarify whether the clinical benefits of *Rhodiola* species are primarily metabolic rather than oxygenation-related. Furthermore, their medical histories should be recorded at baseline, especially regarding the prevalence of cardiovascular, cerebrovascular, and respiratory diseases related to high-altitude hypoxic environments. Furthermore, given that the active constituents of R. rosea overlap mechanistically with food and medicinal homological resources that regulate lipid metabolism via AMPK and PPARγ signaling (Hao et al., 2024), future RCTs should incorporate metabolic biomarkers—such as lipid profiles, glucose homeostasis indicators, and adipokine levels—alongside traditional antioxidant and hypoxic adaptation endpoints. This would help clarify whether the clinical benefits of Rhodiola species are primarily metabolic rather than oxygenation-related. Thirdly, the design and reporting of clinical studies should adhere to international standards, such as registration of research protocols, appropriate randomization and allocation concealment, and blinding methods, to improve research quality.

## 5 Conclusion

This meta-analysis demonstrates that supplementation with Rhodiola species enhances systemic antioxidant capacity (TAC) primarily by bolstering antioxidant reserves, without reducing accumulated lipid peroxidation (MDA) or improving maximal oxygen uptake in healthy adults. Its potential role in alleviating high-altitude deacclimatization symptoms requires validation through larger, demographically diverse trials. Future studies should standardize botanical species and phytochemical content, define optimal dosage ranges (200–600 mg/day for antioxidant effects; >1000 mg/day for potential ergogenic effects), extend intervention duration to ≥8 weeks, and explicitly include female and elderly participants to establish population-specific efficacy and refine the clinical positioning of Rhodiola as a targeted metabolic adaptogen. Additionally, mechanistic investigations into AMPK- and PPARγ-mediated metabolic regulation may help elucidate why Rhodiola species alleviate post-high-altitude deacclimatization symptoms without preventing acute mountain sickness, thereby refining their clinical positioning as metabolic adaptogens rather than acute hypoxic protectants.

## Data Availability

The original contributions presented in the study are included in the article/supplementary material. Further inquiries can be directed to the corresponding author.
